# A KRAS-Associated Signature for Prognostic, Immune and Chemical Anti-Cancer Drug-Response Prediction in Colon Cancer

**DOI:** 10.3389/fphar.2022.899725

**Published:** 2022-06-14

**Authors:** Kangjia Luo, Yanni Song, Zilong Guan, Suwen Ou, Jinhua Ye, Songlin Ran, Hufei Wang, Yangbao Tao, Zijian Gong, Tianyi Ma, Yinghu Jin, Rui Huang, Feng Gao, Shan Yu

**Affiliations:** ^1^ Department of Colorectal Surgery, The Second Affiliated Hospital of Harbin Medical University, Harbin, China; ^2^ Department of Breast Surgery, Harbin Medical University Cancer Hospital, Harbin, China; ^3^ Department of General Surgery, The People’s Hospital of Duerbert Mongolian Autonomous County, Harbin, China; ^4^ Department of Gastrointestinal Surgery, The Affiliated Hospital of Medical School of Ningbo University, Ningbo, China; ^5^ Department of Pathology, The Second Affiliated Hospital of Harbin Medical University, Harbin, China

**Keywords:** KRAS mutation, colorectal cancer, prognostic signature, immune microenvironment, immune/chemotherapy

## Abstract

**Background:** KRAS mutation, one of the most important biological processes in colorectal cancer, leads to poor prognosis in patients. Although studies on KRAS have concentrated for a long time, there are currently no ideal drugs against KRAS mutations.

**Methods:** Different expression analysis and weighted gene coexpression network analysis was conducted to select candidate genes. Log-rank tests and Cox regression picked out the prognostic genes to build a KRAS-related gene prognostic score (KRGPS). A nomogram based on KRGPS was built to predict survival of clinical patients. Comprehensive analysis showed the prognosis, immune microenvironment and response to immune therapy and chemotherapy in KRGPS subgroups.

**Results:** We collected a KRGPS from the set of two genes GJB6 and NTNG1, with low-KRGSP patients having better progression-free survival (PFS). Low KRGPS is correlated with high infiltration of activated NK cells, plasma cells and activated memory CD4 T cells and that these cells benefit more from immune checkpoint inhibitor therapy. However, high KRGPS is associated with high infiltration of activated mast cells, pathways of immune dysregulation and a high ratio of TP53 and KRAS mutations. KRGPS subgroups are also sensitive to chemotherapy differently. A nomogram, established based on the KRGPS and pathological stage, predict 3- and 5-years PFS well.

**Conclusions:** The KRAS-associated score acts as a promising signature to distinguish prognosis, molecular and immune characteristics, and benefits from immune and chemical therapy. These KRAS-associated genes could be promising targets for drug design.

## Introduction

In recent years, the incidence and mortality of colorectal cancer (CRC) have gradually increased, particularly in individuals under 50 years of age. Due to new advances in early diagnosis and treatment strategies, the death rate of CRC has dropped. Nevertheless, it still constitutes the third leading cause of cancer-related death around the world (Miller et al., 2019). Many oncogenes, which play a pivotal role in promoting cancer progression, have been reported to be steadily active in cancer due to genetic alterations. Mutations in the RAS family (KRAS, NRAS, and HRAS) are well-known drivers of CRC, and KRAS mutations are available in CRC patients at the highest frequency among them (Vogelstein et al., 1988; Kim et al., 2020). The presentation of KRAS mutations from CRCs is associated with a worse prognosis than non-KRAS oncogenic ones (Hayama et al., 2019; Wiesweg et al., 2019).

KRAS is activated at the membrane downstream of the epidermal growth factor receptor (EGFR) family, allowing signal transmission from the cell surface to the nucleus and managing many crucial cellular processes (Markman et al., 2010). Studies have highlighted that mutations in KRAS accelerate tumorigenesis (Schwitalla et al., 2013) and critically drive resistance to rapamycin (Hung et al., 2010), MEK inhibition (Haigis et al., 2008), and dietary restriction of serine and glycine (Maddocks et al., 2017). Indeed, KRAS mutation permanently promotes over 10 tumorigenic signaling cascades, especially the mitogen-activated protein kinase (MAPK) pathway and phosphoinositide 3-kinase (PI3K) pathway (Thein et al., 2021). Despite more than 3 decades of research efforts, there are still no effective inhibitors against KRAS for routine clinical practice, prompting the concept that RAS may be undruggable (Papke and Der, 2017). In 2019, there was a breakthrough in which two inhibitors, MRTX849 and AMG 510, successfully targeted KRAS (G12C) mutations in colon adenocarcinomas^1^ (Hallin et al., 2020). However, the duration of response for most patients is still not satisfying, with a median progression-free survival of only 6.3 months shown by the most recent clinical trial data (including 42 patients with colorectal cancer) (Hong et al., 2020). We have not identified ideal targets for the development of drugs against KRAS until today, and more information about KRAS is needed.

Researchers always try to display the landscape of CRC focusing on a specific gene or signaling pathway and ignoring the synergistic effects of others. As a result, drugs based on these findings always show great deficiency in patients. The combination of drugs targeting different motifs has shown great advantages according to the outcome of clinical trials, which is more than a single plus of them (Al-Attar and Madihally, 2020; Rudzińska et al., 2021). Thus, we hypothesized that the KRAS-associated signature gene set may be an ideal target to counterbalance the burden of KRAS mutations. In this study, we identified genes affected by KRAS mutations and established a two-gene signature, which is a robust prognostic biomarker and a potential target for drug design.

## Materials and Methods

### Data Source

Gene expression data and corresponding clinical features of colon adenocarcinomas were downloaded from TCGA for training (https://portal.gdc.cancer.gov/). The profile of gene expression (GSE39582), including 574 samples and matched clinical information, was downloaded from the GEO website for validation (https://www.ncbi.nlm.nih.gov/geo/). Gene IDs were transformed by “clusterProfiler” (Yu et al., 2012), and samples were removed with survival times shorter than 1 month.

### Identification of Differentially Expressed Genes

The KRAS mutation was defined as the presence of missense mutation sites including putative driver and unknown significance. To obtain DEGs between subgroups of colon cancer patients with or without KRAS mutations in the TCGA cohort, the R package “edgeR” was used as the standard comparison model (McCarthy et al., 2012). Sequencing expression was normalized through “TMM”, and the DEG threshold was set at |log2 FC| ≥ 0.585 and *p* < 0.05.

### Identification of Hub Genes

The R package “WGCNA” was used to identify hub genes of DEGs. A soft threshold of 4 was obtained with a RsquaredCut at 0.9. Based on a power of 4, we calculated module eigengenes blockwise from all DEGs in one step. Finally, 7 modules were distinguished by setting the merging threshold function at 0.25. The edges between genes of significantly related modules were used to construct the network with weight >0.2 in Cytoscape (v3.8.2).

### Construction of the KRAS-Related Gene Prognostic Score

The R package “survival” was used to evaluate correlations between the hub gene expression levels and the progression-free survival (PFS) of CRC patients. Genes with *p* value <0.05 were identified. Based on the PFS-related genes (pseudogenes were dropped), Lasso regression was conducted 500 times according to the manual of the R package “glmnet”, and a set of genes that contributed to significant PFS was collected. KRGPS was calculated as the formula:

$$\mathrm{KRGPI}=\sum_{i=1}^{n}\left(\mathrm{Ex}p_{i}\mathrm{*Co}e_{i}\right)$$

“n” represents the number of genes in the model, Exp_i_ represents the expression of gene_i_ and Coe_i_ represents the regression coefficient of gene_i_ determined by multivariate Cox regression.

### Construction of Nomogram

Univariate and multivariate Cox regression was used to determine whether the KRGPS was independent of the clinical characteristics (including age, sex, pathological stage) of patients. Based on the KRGPS and pathological stage, a nomogram was constructed to predict the 3- and 5-PFS. The calibration curves and C-index were used to discriminate the nomogram predicted status and the true survival.

### Analysis of Molecular and Immune Characteristics

To analyze the landscape of gene mutations, information was obtained from the TCGA database, and the quantity and quality of gene mutations were analyzed by the R package “Maftools”. Pieces of information between mRNA and TF and miRNA were obtained from chEA3 (https://maayanlab.cloud/chea3/#top), TargetScanHuman 8.0 (http://www.targetscan.org/vert_80/) and miRDB (http://mirdb.org/). Gene set enrichment analysis (GSEA) was used to probe the signaling pathways on which the differentially expressed genes were concentrated. To evaluate the infiltration of immune cells, CIBERSORT. R (available online at HTTPS://cibersort.stanford.edu/) was conducted according to the expression data of samples. The distribution of immune cells was compared between KRGPS subgroups through the Wilcoxon test. Levels of 33 immune checkpoints were also evaluated in KRGPS subgroups.

### Forecasts of Immunotherapeutic and Chemotherapeutic Response

The Tumor Immune Dysfunction and Exclusion (TIDE) algorithm was managed (online at HTTP://tide.dfci.harvard.edu/) to predict clinical responses to immune checkpoint inhibitors as reported (Jiang et al., 2018; Fu et al., 2020). The R package “pRRophetic” was used to estimate the chemotherapeutic response of each patient based on the levels of transcripts.

### Cell Culture, RNA Extraction and Quantitative Real-Time PCR

Human colon cancer cell lines SW620, HCT116 and HT29 were cultured in RPMI-1640 medium (Gibco, United States) supplemented with 10% fetal bovine serum (FBS, Biological Industries, United States) in a humidified 5% CO_2_ atmosphere at 37 C. All cell lines were purchased from American Tissue Culture Collection (ATCC).

Total RNAs of cells were extracted by TRIzol reagent (Invitrogen, United States) and was reversely transcribed to cDNA via PrimeScript™ RT Master Mix (Takara, Japan). Quantitive real-time PCR was performed by using PowerUp™ SYBR™ Green Master Mix (Applied Biosystems, United States) before loading in StepOne™ Real-Time PCR System (Applied Biosystems). Each reaction was tested in quadruplicate. ACTB was used as the internal reference and the 2^(−ΔΔCt) method was used for evaluating the relative mRNA levels. The sequences of primers were listed below:

Human NTNG1: Forward: 5ʹ- GAG​CAT​CCC​TTG​TGA​GCT​GT -3ʹ, Reverse: 5ʹ- TGA​GGA​CTT​TGG​TGG​AAG​CC -3ʹ;

Human GJB6: Forward: 5ʹ- ACA​CTT​TCA​TCG​GGG​GTG​TC -3ʹ, Reverse: 5ʹ- GCA​GTG​TGT​TGC​AGA​CGA​AG -3ʹ;

Human ACTB: Forward: 5ʹ- GAT​TCC​TAT​GTG​GGC​GAC​GA -3ʹ, Reverse: 5ʹ- AGG​TCT​CAA​ACA​TGA​TCT​GGG​T -3ʹ.

### IHC Staining

30 formalin-fixed, paraffin-embedded (FFPE) tissues were obtained from the Department of Colorectal Surgery, Second Affiliated Hospital of Harbin Medical University. Sections of FFPE tissue (5 um thick) were obtained and deparaffinized with xylene and rehydrated using standard procedures. Tissue sections were incubated with anti-GJB6 (ab200866, Abcam) antibody (1:50) for 3 h at room temperature. Washed again with PBS, the slides were incubated with goat anti-rabbit IgG-HRP for 1 h. At last, slides were treated with 3,3′-diaminobenzidine and counterstained with hematoxylin. The staining intensity of GJB6 immunoreactivity was evaluated by two pathologists respectively.

### DNA Extraction and Sanger Sequencing

DNA was extracted from eight 5 μm FFPE tissues using the Tiangen DNA FFPE Tissue Kit (Tiangen, China) according to the manufacturer’s protocol. To investigate the mutational status of codons 12 and 13, exon 2 of the KRAS gene was amplified with a specific primer: Forward: 5′-ATT​ACG​ATA​CAC​GTC​TGC​AGT​CAA​CTG-3′, Reverse: 5′-CAA​TTT​AAA​CCC​ACC​TAT​A ATGGT-3’. PCR products were purified and sequenced at 3730XL (ABI, United States). The sequence data were analyzed using the SnapGene (Version 6.02).

### Statistical Analysis

All statistical analyses were performed using R software (Version: 4.0.4) and Python (Version: 3.8.8). The Wilcoxon test was performed to compare variables between two groups. *p* value <0.05 was considered significant.

## Results

### KRAS Mutations in CRC Patients

**FIGURE 1:**
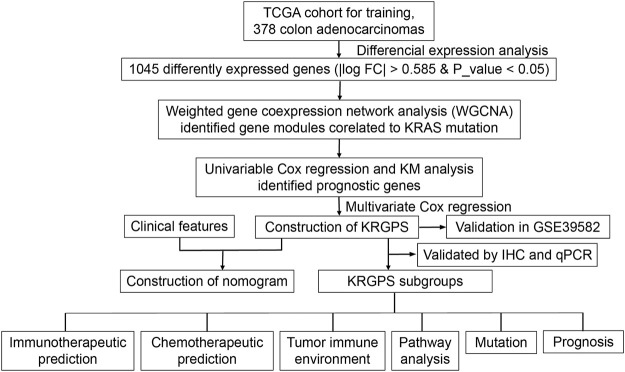
Flow chart overview of the schedule performed to construct a prognostic gene model of colon adenocarcinoma.

CRC treatment decisions have been based on histological considerations for a long time (National Health Commission of the People’s Republic of China, 2020), while novel insights into tumor biology and genetic alterations have rapidly changed the process of therapeutic decisions. KRAS, according to TCGA, is the fourth mutation gene in colon adenocarcinomas (CAs) at a frequency of 37%, with missense as the leading mutation type (Supplementary Figure S1A). Compared with unaltered CAs, KRAS-mutated CAs suffered from shorter disease-free survival and progression-free survival times (Supplementary Figures S1B,C). Then, we conducted our exploration to seek details about KARAS mutations (Figure 1).

### Hub DEGs Related to KRAS Mutation

**FIGURE 2:**
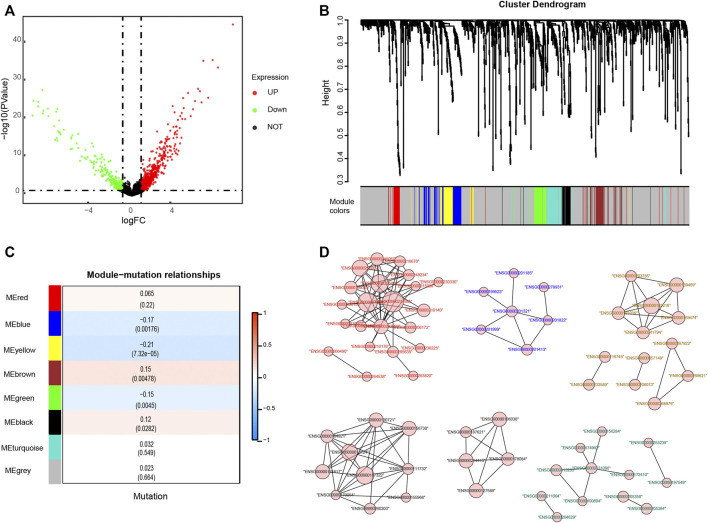
Identification of genes related to KRAS mutations. **(A)** Differentially expressed genes (DEGs) of KRAS mutation subgroups with a cut-off at *p* < 0.05 and |log_2_FC| >0.585 **(B)** Weighted gene coexpression network analysis (WGCNA) of KRAS mutation-related genes with a soft threshold β = 4 **(C)** Spearman correlation analysis of gene modules and KRAS mutations. **(D)** The gene network of KRAS-related genes. The color of the labels and the shape of gene nodes indicate the affiliation of genes.

Depending on the status of KRAS mutation, the samples of colon adenocarcinomas were divided into two subgroups. A total of 1,045 DEGs (|logFC| > 0.585 and *p*-value < 0.05) were obtained, of which 700 genes were upregulated and 345 were downregulated in the KRAS mutation group compared with the KRAS-unaltered group (Figure 2A). Weighted gene coexpression network analysis (WGCNA) was conducted on these DEGs to obtain the hub genes. With a correlation coefficient greater than 0.9, the optimal soft-thresholding power was 4 in terms of the scale-free network (Supplementary Figure S2). Based on the optimal power, the DEGs were apportioned into 7 modules through average linkage hierarchical clustering (Figure 2B and Supplementary Figure S3A). As shown by the Pearson correlation coefficient between a module and sample character, brown and black modules were positively related to KRAS mutation status, while blue, yellow and green modules were negatively related (Figure 2C). Gene network of these modules is shown in Figure 2D. KEGG and GO analyses based on these genes are displayed in Supplementary Figures S3B,C. Negative regulation of megakaryocyte differentiation, antimicrobial humoral response, humoral immune response mediated by circulating immunoglobulin and lymphocyte chemotaxis were core immune processes, on which these genes were concentrated (Supplementary Figure S3D).

### Identification of Prognostic Signatures and Construction of a Nomogram

**FIGURE 3:**
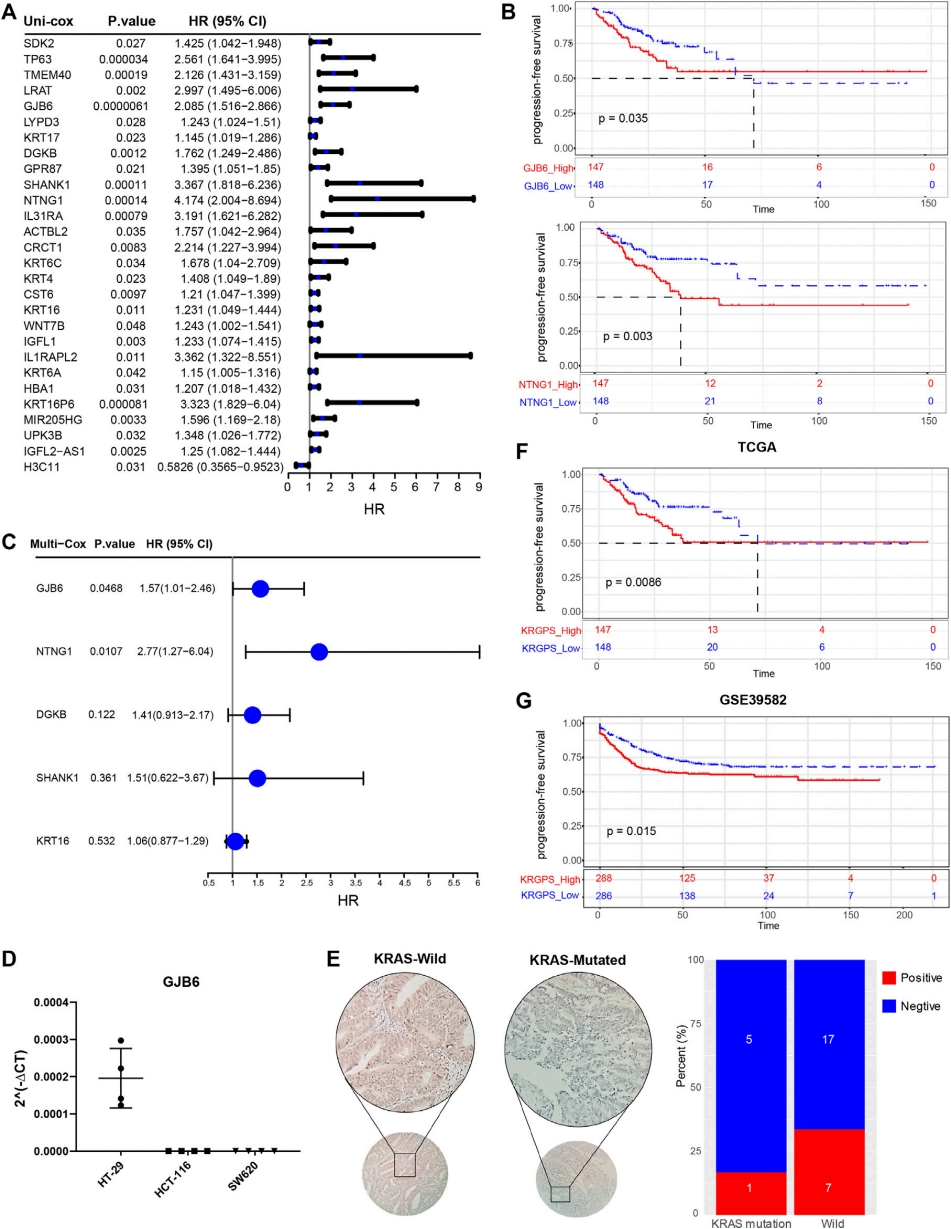
Identification of prognostic signatures. **(A)** Univariate Cox analysis of 28 KRAS-related genes with *p* < 0.05. **(B)** Kaplan–Meier survival analysis of NTNG1 and GJB6 in TCGA cohort (p < 0.05) **(C)** Multivariate Cox regression analysis of 5 prognostic genes determined by Kaplan–Meier survival analysis. **(D)** RT-PCR validation of GJB6 in different COAD cell lines. HT-29 was KRAS-wild while HCT-116 (G13) and SW620 (G12) were KRAS-mutated **(E)** Representive IHC results of GJB6 in CRC patients with KRAS mutated or not. Scale, 200x. Percent of GJB6 positive samples in KRAS-mutated and wild patients. **(F)** Kaplan–Meier survival analysis of KRGPS in the TCGA cohort (Log-rank test) **(G)** Validation of KRGPS in the GEO cohort by Kaplan–Meier survival analysis (Log-rank test).

To determine the prognostic genes, univariate Cox regression and K-M analysis were performed. With *p* < 0.05, 5 genes were regarded as progression-free survival (PFS)-related (Figures 3A,B, Supplementary Figures S4A–C), based on which multivariate Cox regression was conducted before we obtained a set of 2 prognostic genes. As shown in Supplementary Figure S5A, missense mutations were most prevalent in these genes, and their total mutation rates were no more than 4%. For the regulatory network of biomarker genes, there were 2 transcription factors (TFs) and 5 miRNAs interacting with both genes (Supplementary Figure S5B). Next, we evaluated the levels of biomarker genes in colon cancer and normal colon epithelium cell lines. The outcomes of qPCR displayed that GJB6 was expressed in HT-29 cells with wild-type KRAS, while we could hardly detect the levels of it in HCT-116 and SW-620, which is KRAS mutated (Figure 3C). Nor did we detect the expression of NTNG1 in these cell lines (data were not shown). We further evaluated the levels of GJB6 in 30 CRC patients. KRAS mutation at G12/13 site was detected by Sanger sequencing, and 6 of them were KRAS mutated at the site of G12 in exon2 (Supplementary Table S1). To confirm the state of the gene, their levels of GJB6 were evaluated by IHC. Results showed that 7 of 24 were GJB6-positive in KRAS-wild samples, while 1 of 6 samples was positive in KRAS-mutation (Figure 3D).

A KRGPS, with the formula KRGPS = expression of GJB6 * (0.454) + expression of NTNG1 * (1.02), was calculated for prognostic prediction. Samples were separated into low- or high-KRGPS subgroups by the median score. The Kaplan–Meier plot suggested that patients in different KRGPS groups had significantly separated PFS with high KRGPS corresponding to terrible status (Figure 3F). Interestingly, high KRGPS also predicted worse progression-free survival of patients in GSE39582 and shorter overall survival (Figure 3G and Supplementary Figure S4D).

**FIGURE 4:**
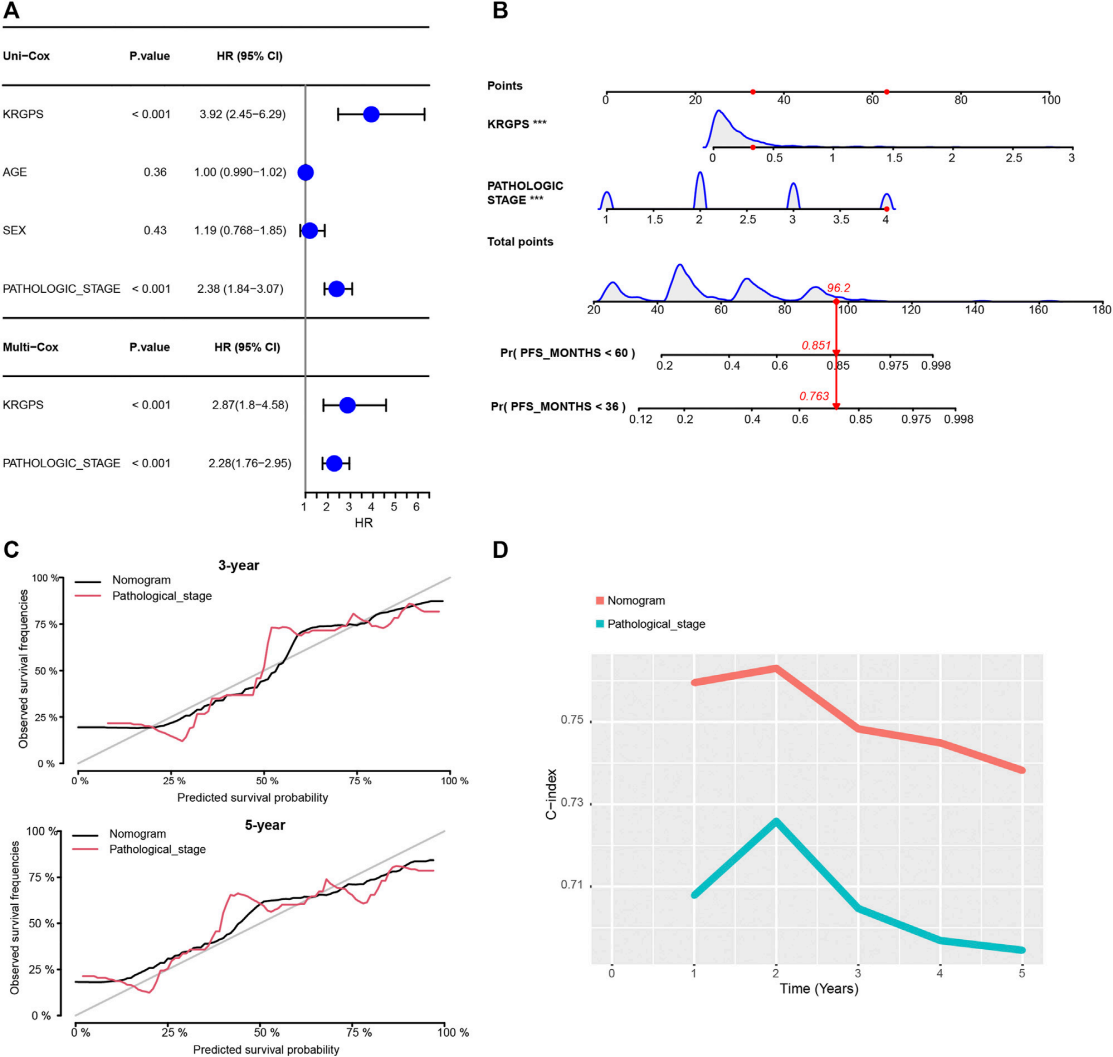
Construction of the nomogram. **(A)** Univariate and multivariate Cox regression of clinical signatures and KRGPS. **(B)** The nomogram was constructed based on the independent prognostic factors evaluated by multivariate Cox regression **(C)** The calibration plots for the internal validation of the nomogram predicting 3-years and 5-years PFS. **(D)** C-index of the nomogram and pathological stage predicting PFS in different years.

Then, we investigated whether the gene-derived risk score was an independent biomarker concerning clinical signatures. Univariate Cox regression analysis showed that KRGPS, as well as pathologic stage, was significantly associated with the prognosis of CRC patients (Figure 4A). Multivariate Cox regression analysis validated KRGPS as an independent prognostic factor adjusted for other clinical signatures (Figure 4A). Based on KRGPS and pathologic stage, a nomogram was constructed to predict the prognosis of CRC patients at 3 and 5 years (Figure 4B). Calibration plots indicated that the nomogram had a good performance and was therefore an ideal model (Figure 4C). The C-index of the nomogram indicated a better prognostic value than the pathological stage, which is recommended as the gold standard for clinical decisions over time (Figure 4D).

### Molecular Characteristics of Different KRGPS Subgroups

**FIGURE 5:**
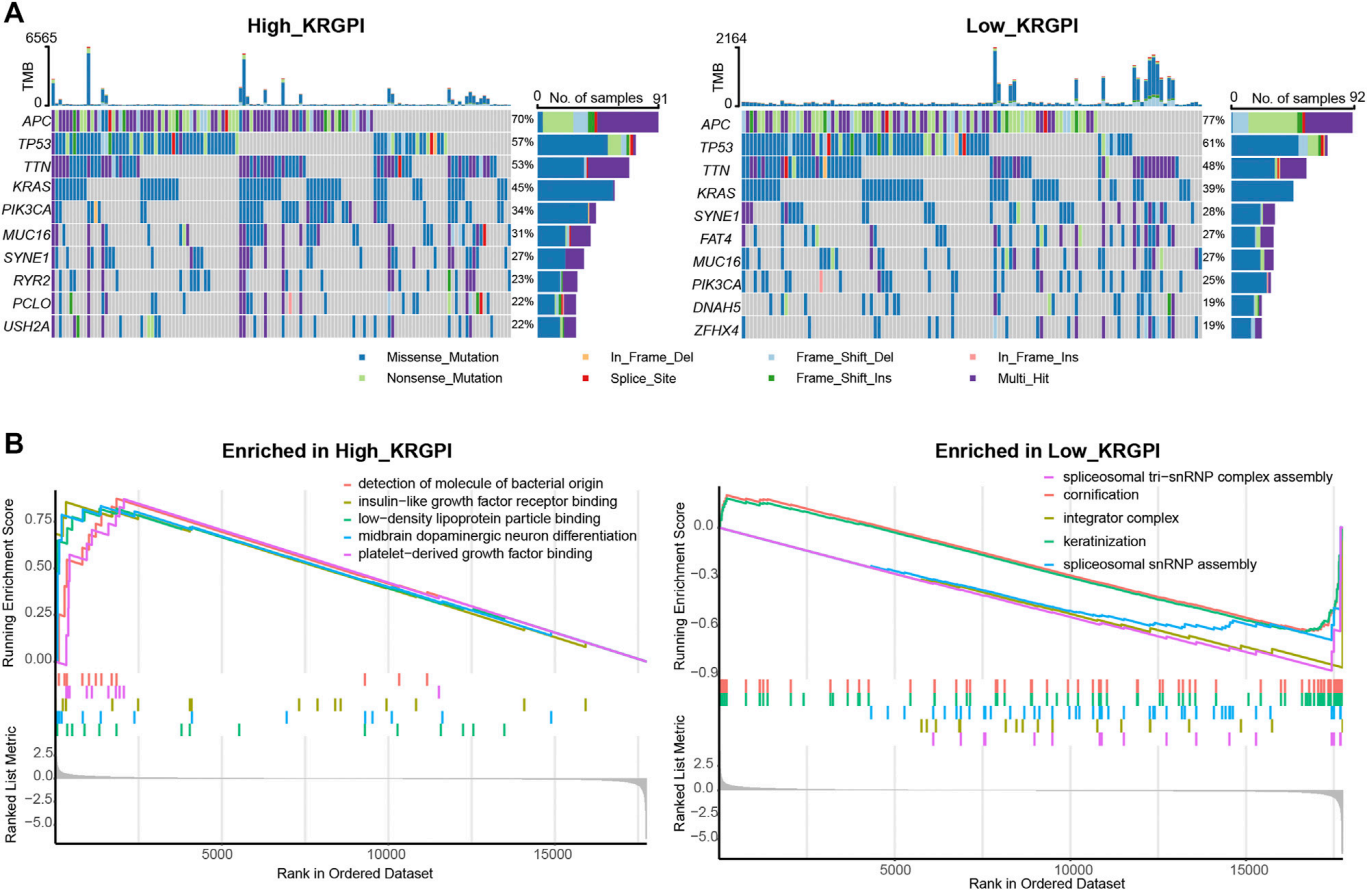
Molecular characteristics of KRGPS subgroups. **(A)** Top 10 mutated genes in KRGPS subgroups. **(B)** Top 5 pathways enriched in different KRGPS subgroups by GO analysis (*p* < 0.05).

Somatic mutations were also evaluated in subgroups of KRGPS with the heading mutated genes APC, TP53, TNN and KRAS in both subgroups (Figure 5A). Mutations in FAT4, DNAH5 and ZFHX4 were more common in the KRGPS-low subgroup, although USH2A, RYR2 and PCLO were more common in the KRGPS-high subgroup (Figure 5A). Fisher’s exact test showed that the mutation status of TTN, PIK3CA, MUC16, SYNE1, RYR2, PCLO and USH2A were correlated to KRGPS in statistics (Supplementary Table S2). Additionally, the TMB of the KRGPS-low subgroup was lower than that of the KRGPS-high subgroup (Supplementary Figure S6A).

The results of GO analysis by GSEA showed that spliceosomal tri-snRNP complex assembly, integrator complex, spliceosomal snRNP assembly, keratinization and cornification were heading functions enriched in low-KRGPS subgroups, while the gene sets of the high-KRGPS subgroups were mainly enriched in functions of detection of molecules of bacterial origin, platelet-derived growth factor binding, insulin-like growth factor receptor binding, midbrain dopaminergic neuron differentiation and low-density lipoprotein particle binding (Figure 5B). KEGG analysis revealed enrichment of malaria, the PI3K-Akt signaling pathway, cytokine–cytokine receptor interaction and the AGE-RAGE signaling pathway in diabetic complications in the KRGPS-high group but pancreatic secretion in the KRGPS-low group (Supplementary Figure S6B).

### Immune Landscape in Different KRGPS Subgroups

**FIGURE 6:**
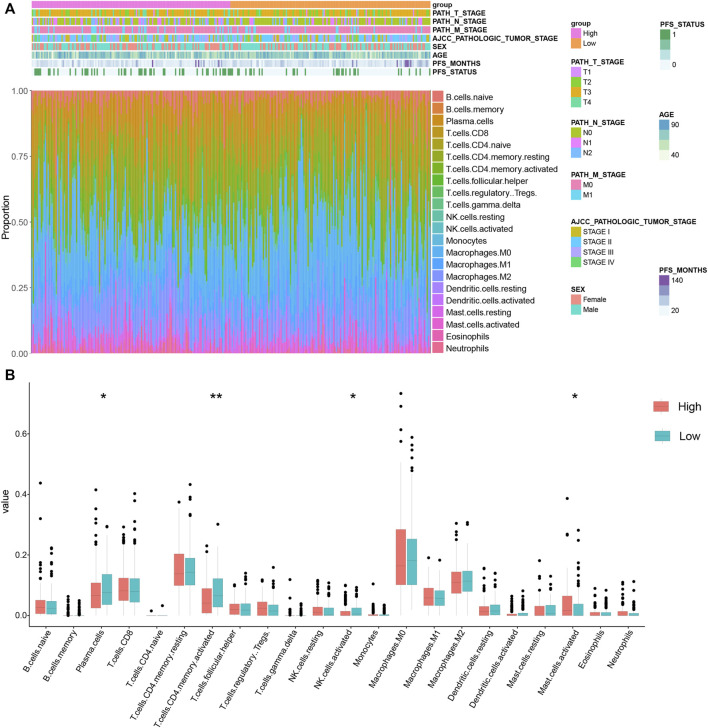
The distribution of infiltrating immune cells in KRGPS subgroups. **(A)** The landscape of the tumor immune environment and clinical features of patients. **(B)** Levels of 22 infiltrated immune cells in KRGPS subgroups by Cibersort. *p < 0.05, **p < 0.01, ***p < 0.001, ****p < 0.0001.

As the GO analysis showed, the KRGPS-related genes were associated with the immune response. A total of 22 types of infiltrating immune cells were evaluated among samples through CIBERSORT. The clinicopathological characteristics of different KRGPS subgroups related to the immune landscape are presented in Figure 6A. Plasma cells, activated NK cells and activated memory CD4 T cells were enriched in the low-KRGPS group, while activated mast cells were concentrated in the high-KRGPS group (Figure 6B). We also observed the relevance between KRGPS and 33 immune checkpoints, of which 20 genes were significantly decreased in the low-KRGPS group compared with the high-KRGPS group (Supplementary Figure S7). The antitumoral immune microenvironment may contribute to the good prognosis of patients in the low-KRGPS subgroup.

### Prediction of Immune and Chemical Therapy in KRGPS Subgroups

**FIGURE 7:**
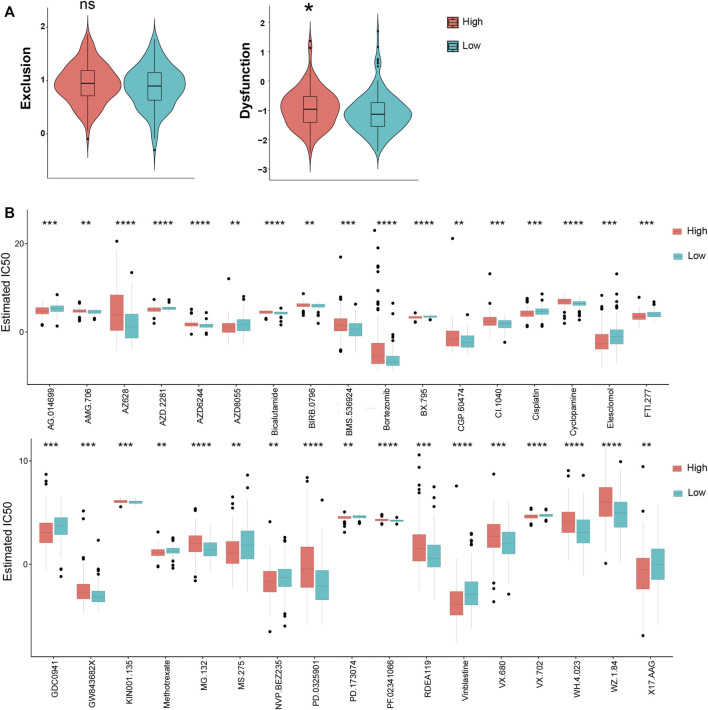
The prognostic value of KRGPS in patients receiving immune and chemical therapy. **(A)** Exclusion and dysfunction scores of samples in KRGPS subgroups. **(B)** Chemotherapeutic responses of high- and low-KRGPS patients. *adj.*p* < 0.05, **adj.*p* < 0.01, ***adj.*p* < 0.001, ****adj.*p* < 0.0001.

TIDE was used to assess the potential clinical efficacy of immunotherapy in different KRGPS subgroups. In this study, the low-KRGPS subgroup corresponding to low dysfunction scores implied that KRGPS-low patients could benefit more from immune checkpoint inhibitor (ICI) therapy than KRGPS-high patients, although the T cell exclusion scores showed no significant differences between the KRGPS subgroup (Figure 7A). Next, we investigated the response to chemotherapy of patients in KRGPS subgroups. A total of 34 drugs displayed significant differences in the estimated IC50 of patients in separated KRGPS subgroups (Figure 7B).

## Discussion

CRC leads to a large number of cancer-related mortalities around the world, and KARAS mutations contribute to a poor prognosis and resistance to receptor tyrosine kinase (RTK) inhibitors and monoclonal antibodies against epidermal growth factor receptor (EGFR) (cetuximab and panitumumab), for example, in CRC patients (Karapetis et al., 2008). More importantly, drugs targeting KRAS directly or indirectly have not been satisfied until today (Zhu et al., 2021). Under this circumstance, more details about KRAS mutations are required, which should be a powerful foundation for drug design.

In our study, WGCNA was first used to recognize the modules of genes correlated to KRAS mutations. Survival analysis indicated a set of four genes that forecast the prognosis of patients efficiently. Based on these genes, we established a KRGPS to predict CRC prognosis and separated patients with CRC into two subgroups, with low-KRGPS patients having a better prognosis. KRGPS was made up of two genes, namely, GJB6 and NTNG1. Results of qPCR suggested that the levels of GJB6 were distinguished according to the status of KRAS-mutation, though we didn’t detect the signals of NTNG1 in colon cancer cell lines. In CRC patients, the GJB6 levels were further evaluated by IHC, as well as the KRAS status by Sanger sequencing. More GJB6 were detected in KRAS-wild patients compared with KRAS-mutated, which validated the findings in colon cell lines. Research had announced that the levels of GJB6 were also decreased during the development of cancers, including gastric cancer, gliomas and head and neck cancer (Ozawa et al., 2007; Sentani et al., 2010; Artesi et al., 2015). For NTNG1, as reported, hypermethylated regions were frequently detected in cancers, implying its potential function for CRC resistance and why there was no detection of this gene (Andrew et al., 2017).

Despite the evolution of intrinsic molecules in tumor cells, the development of cancer has been regarded as a process that leads from cancer immunosurveillance to tumor escape (Dunn et al., 2002). According to the model of immunosurveillance, the clinical manifestation of cancer is dual: 1) neoplastic cell variants with limited immunogenicity are detected by the immune system, and 2) neoplastic cells actively restrain tumor-targeting immune reactions (Pietrocola et al., 2017). Increased immunosuppressive cells, decreased immunoreactive cells and increased expression of immune checkpoints in immune cells and tumors always come along with cancers, especially in advanced patients. In our study, as functional enrichment progressed, genes of WGCNA modules that correlated firmly with KRAS mutations took part in many immune processes. GO analysis revealed that functions, such as regulation of macrophage differentiation, B cell receptor signaling pathway, and regulation of humoral immune response, clustered in patients in the KRGPS subgroup. Next, we compared the distribution of immune cells of subgroups. The enrichment of plasma cells, activated NK cells and activated CD4 memory T cells and the decreased activated mast cells may account for the better survival of low-KRGPS patients. On the other hand, the levels of 18 immune checkpoints were significantly inhibited in the low-KRGPS subgroup, in favor of immune activation. Collectively, KRGPS is a biomarker of active immunity.

Studies on gene mutations also provide insight into the nature of the KRGPS subgroups. A lower incidence of KRAS mutations was located in the low-KRGPS group than in the high-KRGPS group, showing the largest difference in mutations between groups. Moreover, there was a higher rate of TP53 mutation in the high-KRGPS group. TP53 mutation, as reported, determines many biological behaviors of CRC, such as lymphatic and vascular invasion, chemoresistance, and the prognosis of patients (Russo et al., 2005; Iacopetta et al., 2006; Li et al., 2015). In this way, KRGPS-low patients with high KRAS and TP53 mutations had worse survival than KRGPS-high patients, in agreement with our survival outcomes. Tumor mutational burden (TMB), a summary of gene mutations, is positively related to the response of patients receiving immune therapy (Schrock et al., 2019). Our study showed that low-KRGPS patients have low TMB scores, implying a lower likelihood of low-KRGPS patients benefiting from immune checkpoint inhibitors (ICIs).

The tumor immune dysfunction and exclusion (TIDE) module can estimate multiple published transcriptomic biomarkers to predict patient response, which identifies factors that undergo two mechanisms of immune escape: the induction of T cell dysfunction in tumors with high infiltration of cytotoxic T lymphocytes (CTLs) and T cell exclusion in tumors with low CTL levels (Jiang et al., 2018). Low KRGPS, consisting of low T cell dysfunction, may predict a favorable response to ICIs. In contrast, KRGPS predicts the opposite response of ICIs depending on TMB and TIDE. Further studies are calling for, although Liu announced that TIDE predicted the response of ICIs more accurately than other biomarkers, such as mutation load. Chemotherapy is widely used in cancer therapy, and high-KRGPS patients with colon cancer were significantly sensitive to 15 chemotherapeutic agents but blunt to 19 drugs compared with low-KRGPS patients.

In routine clinical guidelines, the pathologic stage is a pivotal prognostic joint of CRC patients. However, it could not fully reflect the biological heterogeneity of patients, as patients with the same pathologic stage lead to absolutely separate outcomes. Currently, the combination of gene markers and clinical signatures is widely used to predict the prognosis of patients. We constructed a nomogram based on the KRGPS derived from genes related to KRAS mutation and pathologic stage to predict patient outcomes, which shows a better efficiency of prediction.

This study still had several limitations. First, our study universally analyzed KRAS mutations, ignoring the separation among molecular subtypes. Mysteries on specific KRAS mutations are waiting for exploration. Moreover, on account of the retrospective data from public databases, case selection bias may cover up some problems. A randomized controlled trial may unravel more information about KRAS mutation.

## Conclusion

Overall, for the first time, this study identifies a two-gene signature associated with KRAS mutations that can independently predict the prognosis of patients with CRC. Gene-derived KRGPS also helps to distinguish immune and molecular features, although further explorations are needed to clarify more details. These two genes, NTNG1 and GJB6, could be potential targets for drug design.

## Data Availability

The datasets presented in this study can be found in online repositories. The names of the repository/repositories and accession number(s) can be found in the article/Supplementary Material.

## Footnotes

1 Author Anonymous (2019). AMG 510 First to Inhibit “Undruggable” KRAS Cancer Discov. 9(8), 988–989. doi:10.1158/2159-8290.CD-NB2019-073
