# Supplementary material for: A KRAS-Associated Signature for Prognostic, Immune and Chemical Anti-Cancer Drug-Response Prediction in Colon Cancer
Source: Front Pharmacol. 2022 Jun 14;13:899725. doi: 10.3389/fphar.2022.899725 (PMC9237412; doi:10.3389/fphar.2022.899725)
Supplement: Supplementary file 1 [file Table1.DOC]

**STable1 Clinical features and KRAS mutation of CRC patients**

| Clinicopathological features | Number |
| --- | --- |
| Gender |  |
| Male | 18 |
| Female | 12 |
| Age (years) |  |
| ≥ 60 | 15 |
| < 60 | 15 |
| T stage |  |
| T1 | 1 |
| T2 | 1 |
| T3 | 27 |
| T4 | 1 |
| N stage |  |
| N0 | 17 |
| N1 | 7 |
| N2 | 6 |
| KRAS mutation |  |
| G12 | 6 |
| Wild | 24 |
| Total | 30 |
