# Supplementary material for: A KRAS-Associated Signature for Prognostic, Immune and Chemical Anti-Cancer Drug-Response Prediction in Colon Cancer
Source: Front Pharmacol. 2022 Jun 14;13:899725. doi: 10.3389/fphar.2022.899725 (PMC9237412; doi:10.3389/fphar.2022.899725)
Supplement: Supplementary file 2 [file Table2.DOC]

**STable2 Fisher’s exact test of gene mutation and KRGPS**

| **Gene_ID** | ***P*_value** |
| --- | --- |
| TP53 | 0.913543228 |
| KRAS | 0.095452274 |
| SMAD4 | 0.195902049 |
| APC | 0.336331834 |
| TTN | 0.00049975 |
| PIK3CA | 0.048975512 |
| MUC16 | 0.0009995 |
| SYNE1 | 0.021489255 |
| RYR2 | 0.006996502 |
| PCLO | 0.009995002 |
| USH2A | 0.00049975 |
| FAT4 | 0.821589205 |
| DNAH5 | 0.211894053 |
| ZFHX4 | 0.092453773 |
